# Rosuvastatin, but not atorvastatin, enhances the antihypertensive effect of cilostazol in an acute model of hypertension

**DOI:** 10.1007/s00210-023-02758-1

**Published:** 2023-10-11

**Authors:** Ahmed Hamdy, Hany M. El-Bassossy, Shimaa M. Elshazly, Shaimaa S. El-Sayed

**Affiliations:** Department of Pharmacology and Toxicology, Faculty of Pharmacy, Zagazig University, Zagazig, 44519 Egypt

**Keywords:** Statins, Cilostazol, Baroreflex sensitivity, Hypertensive emergency

## Abstract

**Purpose:**

Hypertensive emergency, a sudden and severe increase in blood pressure, necessitates immediate intervention to avoid end-organ damage. Cilostazol, a selective phosphodiesterase-III inhibitor, has vasodilator effect. Here, we investigated the effect of two commonly used statins, atorvastatin or rosuvastatin, on cilostazol antihypertensive activity in acute model of hypertension.

**Methods:**

Hypertensive emergency was induced via angiotensin II intravenous infusion (120 ng.kg^−1^.min^−1^). Rats were subjected to real-time arterial hemodynamics and electrocardiogram recording while investigated drugs were injected slowly at cumulative doses 0.5, 1, and 2 mg.kg^−1^, individually or in combination, followed by baroreflex sensitivity (BRS) analysis and serum electrolytes (Na^+^ and K^+^) and vasomodulators (norepinephrine (NE), and nitric oxide (NO)) assessment.

**Results:**

Cilostazol reduced systolic blood pressure (SBP), while co-injection with rosuvastatin augmented cilostazol SBP-reduction up to 30 mmHg. Compared to atorvastatin, rosuvastatin boosted the cilostazol-associated reduction in peripheral resistance, as evidenced by further decrease in diastolic, pulse, and dicrotic-notch pressures. Rosuvastatin co-injection prevented cilostazol-induced changes of ejection and non-ejection durations. Additionally, rosuvastatin coadministration produced better restoration of BRS, with an observed augmented increase in BRS indexes from spectral analysis. Greater reduction in sympathetic/parasympathetic ratio and serum NE upon rosuvastatin coadministration indicates further shift in sympathovagal balance towards parasympathetic dominance. Additionally, rosuvastatin coinjection caused a greater decrease in serum sodium, while more increase in NO indicating augmented reduction of extracellular volume and endothelial dysfunction.

**Conclusion:**

Rosuvastatin boosted cilostazol’s antihypertensive actions through effects on peripheral resistance, BRS, sympathovagal balance, endothelial dysfunction, and electrolytes balance, while atorvastatin did not demonstrate a comparable impact.

## Introduction

Hypertension is a major risk factor for life-threatening conditions such as cardiovascular diseases, stroke, kidney disease, and peripheral arterial diseases (Fuchs and Whelton 2020; Makin et al. 2001). Thirty-one percent of the world’s population suffers from hypertension (Bloch 2016; Mills et al. 2016). Hypertensive emergency is defined as an acute elevation in blood pressure (BP) over 180/120 mmHg usually associated with signs of target-organ damage and affects about 1–2% of hypertensives during their lifespan (Alley and Copelin 2018; van den Born et al. 2019). The treatment of hypertensive emergency requires reduction of mean arterial pressure by 20–25% in the first hour to alleviate end-organ dysfunction. This is usually achieved via intravenous infusion of direct vasodilators such as sodium nitroprusside, nitroglycerine, hydralazine, and nicardipine (van den Born et al. 2019). Although most vasodilators work effectively, they can cause reflex tachycardia which may attenuate their antihypertensive activity (Aggarwal and Khan 2006).

Baroreflex sensitivity (BRS) is one of the most important physiological buffering mechanisms that contributes to maintaining heart rate, myocardial contractility, conductivity, vascular resistance, and consequently leading to BP homeostasis (La Rovere et al. 2011). Both chronic hypertension and hypertensive emergency have been associated with disturbed BRS and sympathovagal imbalance (de Queiroz et al. 2013; Lagi and Cencetti 2015; Valensi 2021). Restoring BRS, heart rate variability (HRV), and sympathovagal balance is thought to be a viable approach to managing hypertension (de Leeuw et al. 2017; Navaneethan et al. 2009).

Cilostazol, a selective phosphodiesterase-III enzyme inhibitor, is well-known for vasodilator and antiplatelet activity and is frequently used for peripheral arterial diseases management (Reddy et al. 2018). Several cardiovascular benefits have been reported upon cilostazol treatment including prevention of either pulmonary arterial hypertension, fatal arrhythmia, or tachycardia-induced atrial remodeling (Sun et al. 2009; Tsuchiya et al. 2002; Zhao et al. 2016). Moreover, in angiotensin II (AnII) hypertensive mice, it effectively decreased elevated systolic blood pressure and alleviated heart failure with diastolic dysfunction (Reddy et al. 2018).

Hypertension and hyperlipidemia are two potential cardiovascular disease risk factors that often coexist (Borghi et al. 2022). Statins, the most prescribed hypolipidemic agents, have other non-hypolipidemic pleiotropic healthy effects that could further reduce cardiovascular risks (Lin et al. 2022). For instance, they have been reported to exert a BP lowering effect (Bautista 2009; Borghi et al. 2001). Atorvastatin lowered systolic and diastolic BP in norm-lipemic patient (Kuklinska et al. 2010). Similarly, rosuvastatin has been reported to reduce BP and attenuate peripheral resistance in hypertensive rats, independently on cholesterol levels (Li et al. 2011; Susic et al. 2003).

Statins’ potentials to enhance cilostazol antihypertensive effect in hypertensive emergency remain uninvestigated, and this was the first aim of this study. The second aim was to scrutinize the means via which statins, particularly atorvastatin and rosuvastatin, may exert their potentiation on cilostazol effect utilizing arterial hemodynamics, cardiac conductivity monitoring techniques as well as serum biochemical measurements in acute model of AnII-induced hypertensive emergency in rats. The impact on peripheral resistance, heart workload, spontaneous BRS, HRV, sympathovagal balance as well as serum vasomodulators (norepinephrine (NE) and nitric oxide (NO)), and electrolytes (Na^+^/K^+^ balance) were investigated.

## Materials and method

### Drugs and chemicals

Atorvastatin and rosuvastatin were kindly provided from Delta Pharma and Mash Premiere, respectively (10th of Ramadan, Egypt), cilostazol, angiotensin II, polyethylene glycol (PEG) 400, and Tween® 80 were purchased from Sigma Aldrich (Munich, Germany). Linoleic acid was purchased from Acros organics (Fair Lawn, New Jersey). Ketamine was purchased from Sigma pharmaceutical industries (Menoufia, Egypt) while xylazine was purchased from ADWIA pharmaceutical industries (10th of Ramadan, Egypt). Linoleic acid–based vehicle was used to prepare the investigated drugs (statins and cilostazol) in the form of self-nanoemulsifying drug delivery system for intravenous injection (Abdallah et al. 2021).

### Animals

Adult male Wistar rats (200–250 g), 6–8 weeks old, were obtained from the Organization for Biological Products and Vaccines of Egypt (Cairo, Egypt). Rats were acclimatized before experiments for 2 weeks. The regulated temperature (23 ± 2 °C), humidity (60 ± 10%), light/dark cycle (12/12 h), and free access to rodent pellet food and water were maintained for animals.

All experimental procedures and animal handling were carried out in accordance with the agreed guidelines for the treatment and use of laboratory animals approved by the Animal Ethics Committee of Zagazig University (approval number: ZU-IACUC/3/F/160/2019).

### Acute hypertension induction

Following a 10-min stabilization period of basal recording of invasive BP and electrocardiogram (ECG), AnII was slowly administered at 120 ng.min^−1^.kg^−1^ through the cannulated femoral vein via digital infusion syringe pump (Advance Infusion system Series 1200, Cell Point Scientific, Gaithersburg.MD) and continued throughout the experiment duration (Abdallah et al. 2021).

### Experimental design

Hypertensive animals were divided into seven groups (*n* = 6/group): Group 1: saline served as time control, Group 2: vehicle (composed of 10% linoleic acid, 80% PEG 400 and 10% tween 80), group 3: atorvastatin (Ator), group 4: rosuvastatin (Rosv), group 5: cilostazol (Clio), group 6: atorvastatin plus cilostazol (Ator + Cilo), and group 7: rosuvastatin plus cilostazol (Rosv + Cilo). After a steady state of elevated BP was achieved, saline, vehicle, and drugs were administered slowly (over 1 min) into the femoral vein at cumulative doses of 0.5, 1, and 2 mg.kg^−1^, 10 min apart, each in 100, 100, and 200 μL injection volume respectively. Invasive BP and ECG were continuously recorded after each dose injection for 10 min.

### Invasive blood pressure monitoring

Animals were anesthetized by intraperitoneal injection of 100 mg.kg^−1^ ketamine and 10 mg.kg^−1^ xylazine. A rectal probe and automatic heating pads were used to keep the rats’ body temperature at 37 °C. Real time arterial BP hemodynamic recording was achieved via a micro-tip pressure–volume catheter (Millar Instruments, Houston, TX, USA) (Ahmed et al. 2021). The catheter was inserted into the right carotid artery through a small opening that continuously monitored arterial BP. Via a power lab data interface, the catheter was connected to a computer running the professional software lab chart (version.8, AD Instruments, Bella Vista, Australia), including BP module (El-Bassossy et al. 2018). In addition, the BP module monitors all hemodynamic parameters including mean arterial pressure (MAP), SBP, diastolic blood pressure (DBP), pulse pressure, dicrotic-notch pressure, and systolic-dicrotic pressure (SDP) difference (Ahmed et al. 2021).

### Electrocardiogram recording

The standard surface ECG was recorded using the power lab system (v8.0, AD Instruments, Bella Vista, Australia) which was connected to a computer with running lab chart professional software (version 8). This software includes an ECG module that detects and quantitatively computes various components of the ECG such as heart rate, ejection duration, and non-ejection duration (El-Bassossy et al. 2018).

### Baroreflex sensitivity analysis

Nevrokard small animal-baroreflex sensitivity, a newly developed software (Medistar, Ljubljana, Slovenia), was utilized for the analysis of spontaneous BRS (Shaltout and Abdel-Rahman 2005). Baroreflex sensitivity was assessed by spectral domain indexes (square root of the ratio of beat-to-beat interval, RRI, and systolic arterial pressure, SAP, in high frequency range [HF-α] and square root of the ratio of RRI and SAP in low frequency range [LF-α]) and by sequential domain method as total sequences of at least three beats in which SAP consecutively increases or decreases (seq BRS-SAP TOTAL) (Shaltout and Abdel-Rahman 2005).

Sympathovagal balance was evaluated by spectral domain method as ratio of power of RRI spectra in low frequency range to that in high frequency range (LF_RRI_/HF_RRI_). Whilst HRV was measured by time domain method as standard deviation of beat-to-beat interval (SDRR) and root mean square of successive differences (rMSSD) (Shaltout and Abdel-Rahman 2005).

### Blood sampling and serum preparation

Blood was collected directly from the femoral vein of rats, allowed to stand for 20 min at 4 °C to allow coagulation, then centrifuged at 5000 × *g* at 4 °C for 15 min, and serum was then aspirated and stored at − 80 °C for biochemical measurements.

### Determination of serum nitric oxide, norepinephrine, sodium, and potassium

Serum NO was assessed by rat ELISA kit (MyBioSource, San Diego, CA, USA) while serum NE were measured using rat ELISA kit supplied by Eagle Biosciences (Nashua, NH, USA). In addition, colorimetric kits from Spectrum Egyptian Co. for biotechnology (Cairo, Egypt) were used to measure serum sodium and potassium. All measurements were conducted as per manufacturers’ instructions.

### Statistical analysis

All values are presented as a mean ± SEM. GraphPad prism Software (version.9, Inc, La Jolla, CA, USA) was used for statistical analysis. Paired student *t* test, one-way ANOVA, and repeated-measures two-way ANOVA followed by Bonferroni’s post hoc test were used for comparisons. The statistical significance was tested at *p* < 0.05*.*

## Results

### Effect on BP (SBP, DBP, and MAP)

**Fig. 1:**
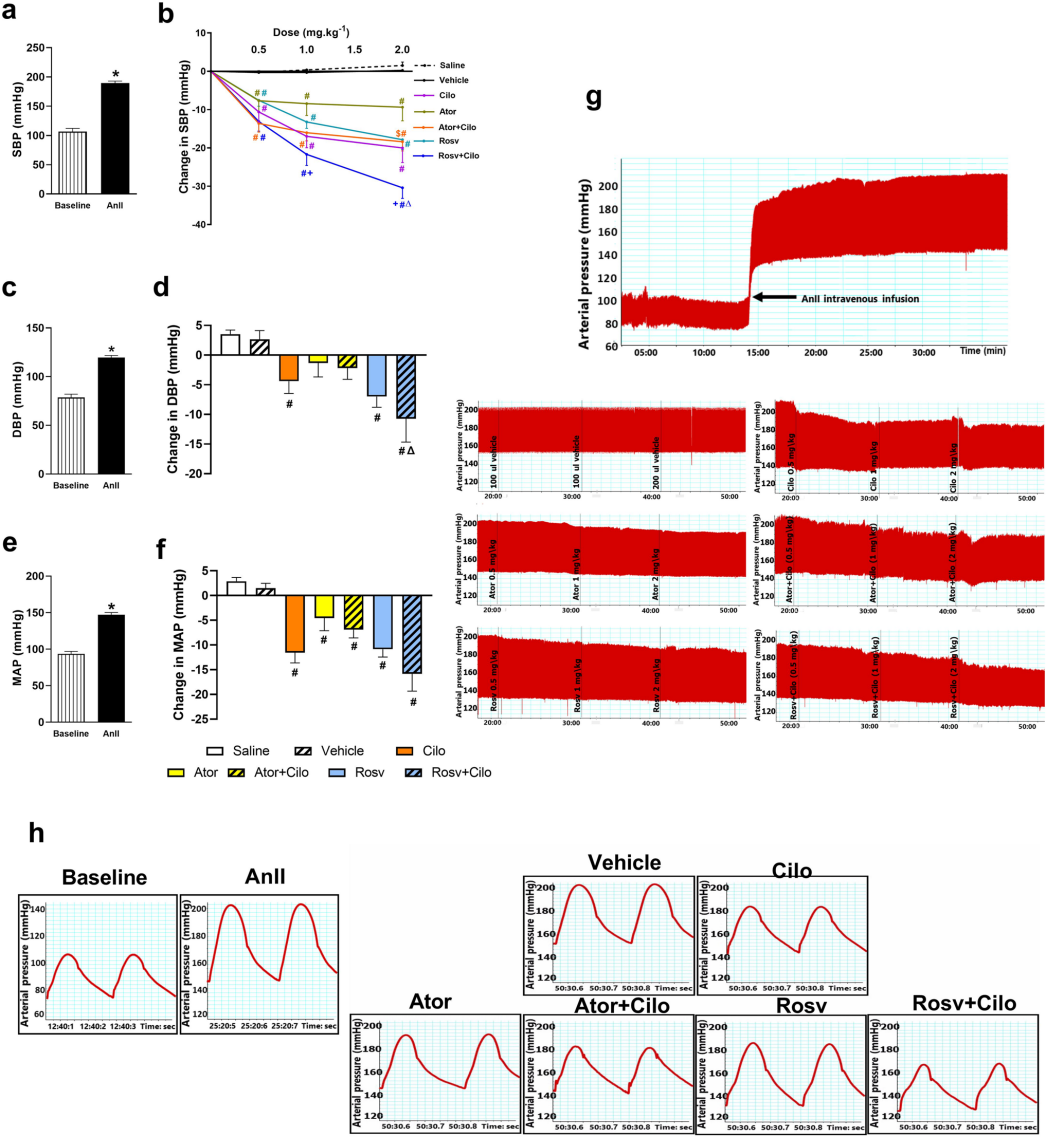
Effect of acute hypertension induced by intravenous infusion of angiotensin II (AnII) at 120 ng.min^−1^.kg^−1^ on systolic blood pressure (SBP, **a**), diastolic blood pressure (DBP, **c**), and mean arterial pressure (MAP, **e**) of adult male Wistar rats and the impact of atorvastatin (Ator) and rosuvastatin (Rosv) on cilostazol (Cilo) effect on elevated SBP (**b**) of hypertensive rats following slow intravenous injection of Cilo either alone or in combination with Ator or Rosv, at successive doses (0.5, 1, and 2 mg.kg^−1^), 10 min apart. Also shown the impact of slow intravenous injection of Ator (2 mg.kg^−1^) and Rosv (2 mg.kg^−1^) on Cilo (2 mg.kg^−1^) effect on DBP (**d**) and MAP (**f**). Values are presented as mean ± SEM (*n* = 6/group). ^*^p < 0.05 vs. baseline, ^*#*^*p* < 0.05 vs. vehicle treated group, ^*Δ*^*p* < 0.05 vs. Cilo-treated group, ^+^*p* < 0.05 vs. Rosv-treated group, and ^*$*^*p* < 0.05 vs. Ator-treated group using paired Student *t* test, one-way ANOVA, and two-way ANOVA followed by Bonferroni post hoc test. Representative images for real time invasive blood pressure (BP) recording (**g**) are shown. Representative traces from BP recording of baseline and following slow intravenous injection of 2 mg.kg^−1^ of each drug and intravenous infusion of AnII (**h**) are shown

Intravenous infusion of AnII at 120 ng.min^−1^.kg^−1^ caused significant elevation of SBP, DBP, and MAP as compared to baseline recordings (Fig. 1a, c, and e). As shown in Fig. 1b, cilostazol, atorvastatin as well as rosuvastatin exhibited a significant dose-dependent reduction of AnII-induced elevations in SBP compared to vehicle treated group at successive doses of 0.5, 1, and 2 mg.kg^−1^. Rosuvastatin co-injection with cilostazol significantly lowered SBP at the three successive doses. Interestingly, rosuvastatin enhanced the SBP lowering effect of cilostazol at 2 mg.kg^−1^ compared to cilostazol alone demonstrating the most SBP lowering effect at the highest dose even when compared to rosuvastatin alone. On the other hand, co-injection of cilostazol with atorvastatin significantly lowered SBP at the three doses compared to vehicle group showing a much lower effect at the highest dose compared to atorvastatin treatment, yet comparable to cilostazol alone no significant difference was noticed.

Compared to vehicle-treated group and following the intravenous injection of the highest dose (2 mg.kg^−1^), cilostazol and both statins significantly lowered AnII-induced elevation in MAP, whereas rosuvastatin and cilostazol, but not atorvastatin, significantly reduced AnII-induced elevation in DBP. Rosuvastatin co-injection enhanced cilostazol lowering effect of DBP as compared to cilostazol treated group (Fig. 1d and f).

### Effect on peripheral resistance indexes (pulse pressure, dicrotic-notch pressure, and SDP difference)

**Fig. 2:**
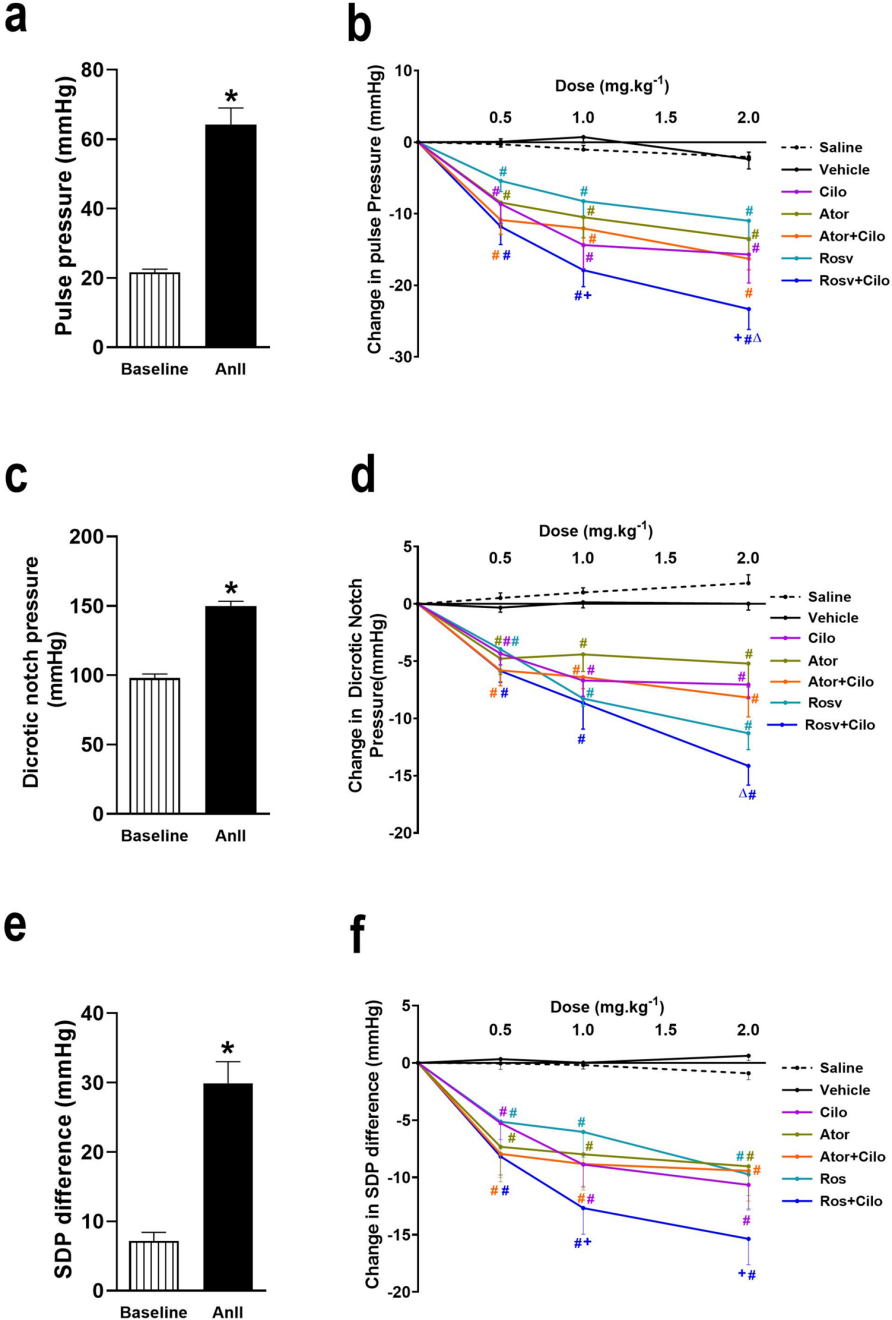
Effect of acute hypertension induced by intravenous infusion of angiotensin II (AnII) at 120 ng.min^−1^.kg^−1^ on pulse pressure (**a**), dicrotic-notch pressure (**c**), and systolic blood pressure-dicrotic notch pressure (SDP) difference (**e**) of adult male Wistar rats and the impact of atorvastatin (Ator) and rosuvastatin (Rosv) on cilostazol (Cilo) effect on pulse pressure (**b**), dicrotic-notch pressure (**d**), and SDP difference (**f**) of hypertensive rats following slow intravenous injection of Cilo either alone or in combination with Ator or Rosv at successive doses (0.5, 1, and 2 mg.kg^−1^), 10 min apart. Values are presented as mean ± SEM (*n* = 6/group). ^*^p < 0.05 vs. baseline, ^*#*^*p* < 0.05 vs. vehicle-treated group, ^*Δ*^*p* < 0.05 vs. Cilo-treated group, and ^+^*p* < 0.05 vs. Rosv-treated group using paired Student *t* test and two-way ANOVA followed by Bonferroni post hoc test

Intravenous infusion of AnII at 120 ng.min^−1^.kg^−1^ caused significant elevation of pulse pressure, dicrotic-notch pressure, and SDP difference as compared to baseline recordings (Fig. 2a, c, and e). As presented in Fig. 2b, d, and f, either cilostazol or both statins significantly reduced AnII-induced augmentation of either pulse, dicrotic-notch pressures, and SDP difference following slow intravenous injection of all three doses respectively (0.5, 1, and 2 mg.kg^−1^). Interestingly, co-injection of rosuvastatin boosted the lowering effect of cilostazol on pulse pressure and dicrotic-notch pressure at the highest dose when compared to cilostazol alone (Fig. 2b and d), however atorvastatin caused no further reduction of peripheral resistance indexes when co-injected with cilostazol. Moreover, rosuvastatin lowering effect of pulse pressure and SDP difference was enhanced on cilostazol-coadministration at the second and third doses when compared to rosuvastatin (Fig. 2b and f).

### Effect on heart rate, ejection duration, and non-ejection duration

**Fig. 3:**
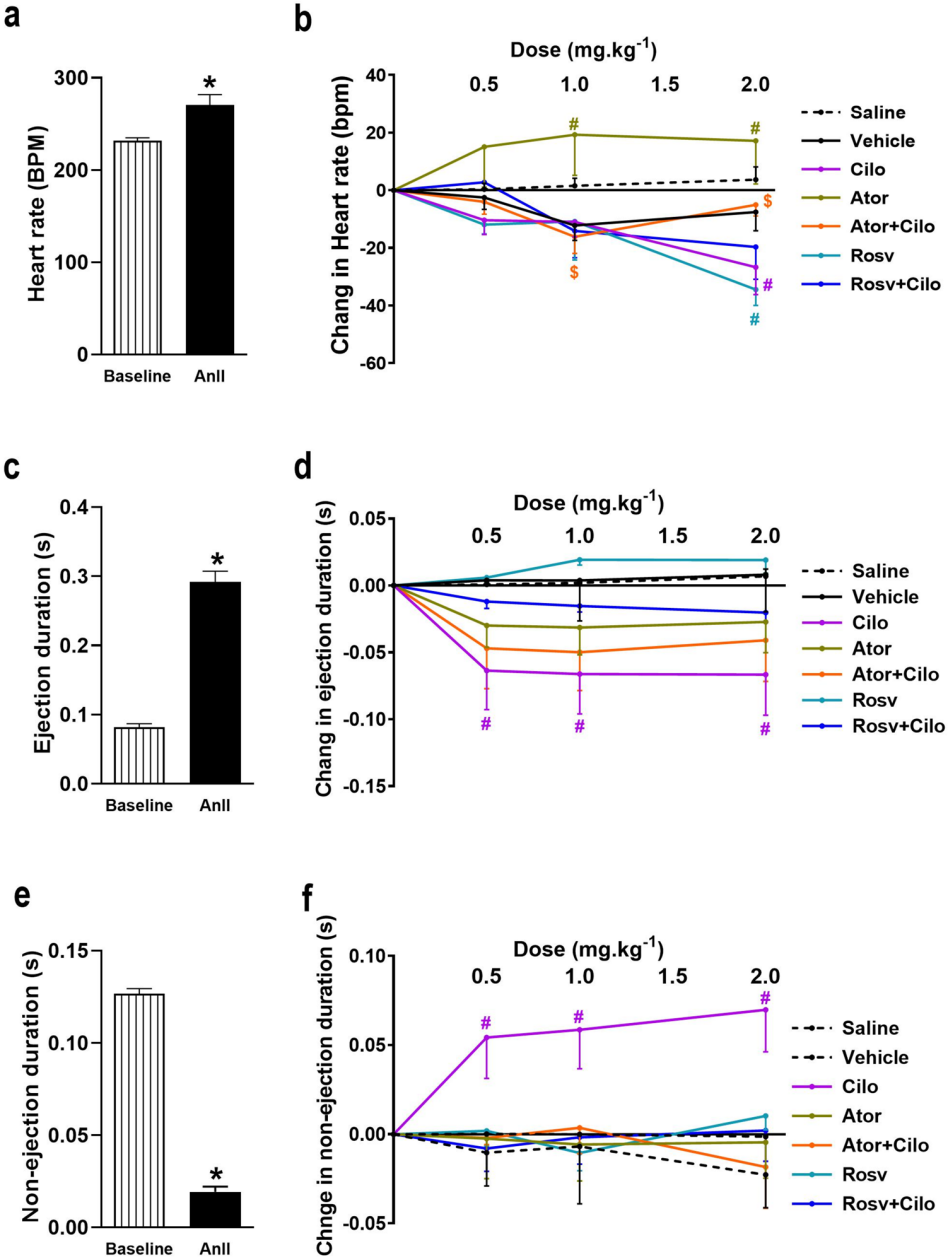
Effect of acute hypertension induced by intravenous infusion of angiotensin II (AnII) at 120 ng.min^−1^.kg^−1^ on heart rate (**a**), ejection duration (**c**), and non-ejection duration (**e**) of adult male Wistar rats and the impact of atorvastatin (Ator) and rosuvastatin (Rosv) on cilostazol (Cilo) effect on heart rate (**b**), ejection duration (**d**), and non-ejection duration (**f**) of hypertensive rats following slow intravenous injection of Cilo either alone or in combination with Ator or Rosv at successive doses (0.5, 1, and 2 mg.kg^−1^), 10 min apart. Values are presented as mean ± SEM (*n* = 6/group). ^*^p < 0.05 vs. baseline, ^*#*^*p* < 0.05 vs. vehicle-treated group, and ^*$*^*p* < 0.05 vs. Ator-treated group using paired Student *t* test and two-way ANOVA followed by Bonferroni post hoc test

Compared to baseline recordings, AnII infusion at 120 ng.min^−1^.kg^−1^ caused significant increase in heart rate, ejection, and non-ejection durations while caused a significant decrease in non-ejection duration (Fig. 3a, c, and e). As displayed in Fig. 3b, effects of different treatment on heart rate were divergent where on comparison with vehicle-treated group both cilostazol and rosuvastatin individually exhibited a significant bradycardia after the third dose while atorvastatin treatment exhibited a pronounced tachycardia at the second and the third doses. Co-injection of cilostazol reversed atorvastatin induced tachycardia at both 1 and 2 mg.kg^−1^ compared to atorvastatin group (Fig. 3b). Furthermore, only cilostazol significantly reduced ejection duration while increased non-ejection duration at all doses compared to vehicle treated group. Noteworthy, rosuvastatin co-injection prevented cilostazol-induced changes of both ejection and non-ejection durations (Fig. 3d and f).

### Effect on baroreflex sensitivity

**Fig. 4:**
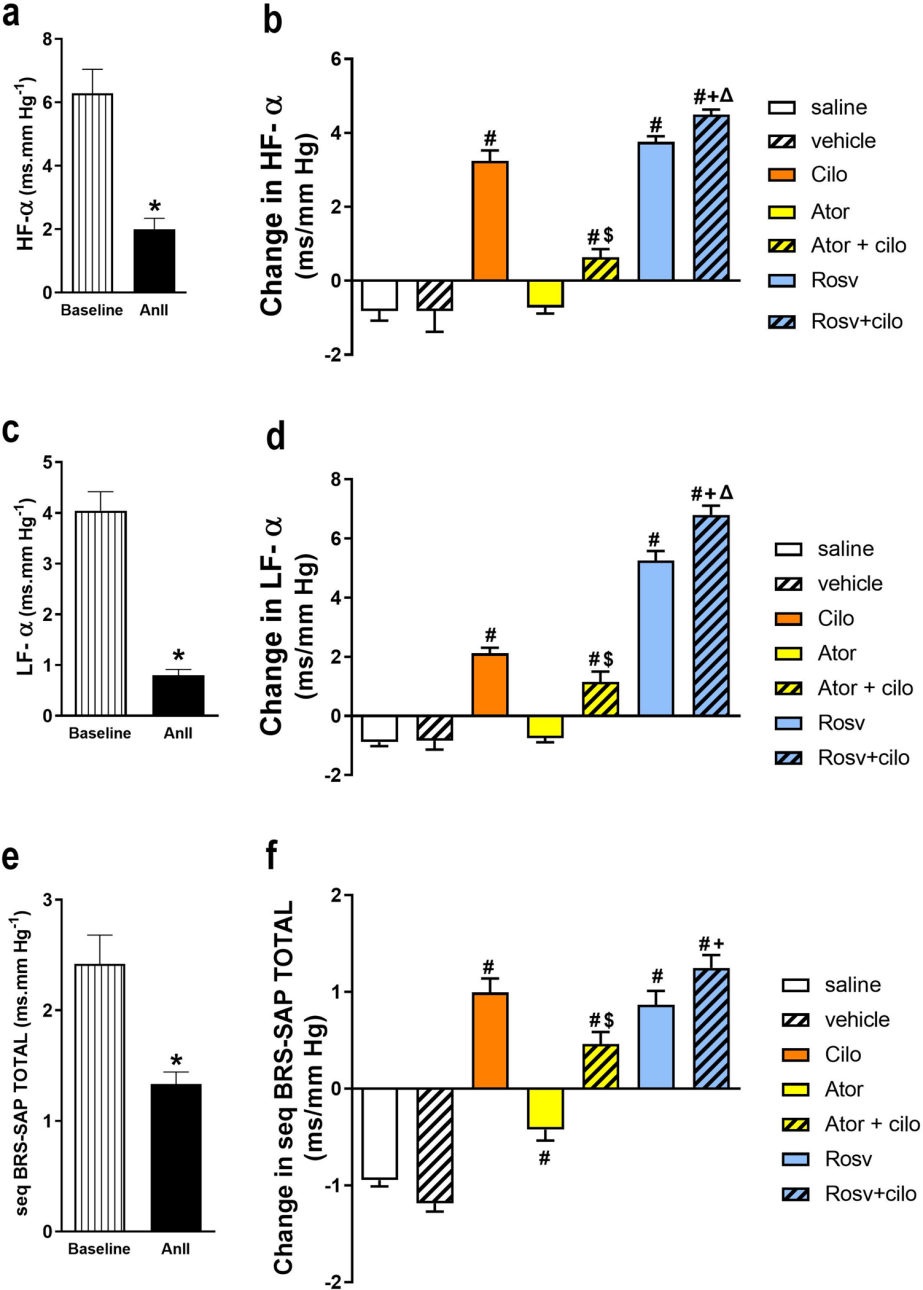
Effect of acute hypertension induced by intravenous infusion of angiotensin II (AnII) at 120 ng.min^−1^.kg^−1^ on baroreflex sensitivity demonstrated by spectral domain indexes; square root of the ratio of RRI and SAP in high frequency range (HF-α, **a**), square root of the ratio of RRI and SAP in low frequency range (LF-α, **c**), and total sequences of at least three beats in which SAP consecutively increases or decreases accompanied by changes in the RRI of the subsequent beats in the same direction (seq BRS-SAP TOTAL, **e**) in the sequential domain of adult male Wistar rats. Impact of slow intravenous injection of atorvastatin (Ator, 2 mg.kg^−1^) and rosuvastatin (Rosv, 2 mg.kg^−1^) on cilostazol (Cilo, 2 mg.kg^−1^) effect on HF-α (**b**), LF-α (**d**), and seq BRS-SAP TOTAL (**f**) of hypertensive rats. Values are presented as mean ± SEM (*n* = 6/group). ^*^p < 0.05 vs. baseline, ^*#*^*p* < 0.05 vs. vehicle-treated group, ^*Δ*^*p* < 0.05 vs. Cilo-treated group, ^*$*^*p* < 0.05 vs. Ator-treated group, and.^+^*p* < 0.05 vs. Rosv-treated group using paired Student *t* test and one-way ANOVA followed by Bonferroni post hoc test. SAP (systolic arterial pressure), RRI (beat-to-beat interval)

To evaluate BRS in different groups, spectral and sequential domain parameters were determined from hemodynamic recordings. As presented in Fig. 4a, c, and e, AnII intravenous infusion (120 ng.kg^−1^.min^−1^) caused reduction of BRS, as demonstrated by significant decrease in HF-α, LF-α, and seq BRS-SAP TOTAL compared to baseline recordings. Following the 2 mg.kg^−1^ dose and comparable to vehicle treated group, cilostazol resulted in significant increase in HF-α, LF-α, and seq BRS-SAP TOTAL compared to vehicle-treated group (Fig. 4b, d, and f)**.** Unlike atorvastatin, rosuvastatin co-injection augmented cilostazol increasing effect in both HF-α and LF-α, compared to cilostazol alone (Fig. 4b and d).

### Effect on heart rate variability

**Fig. 5:**
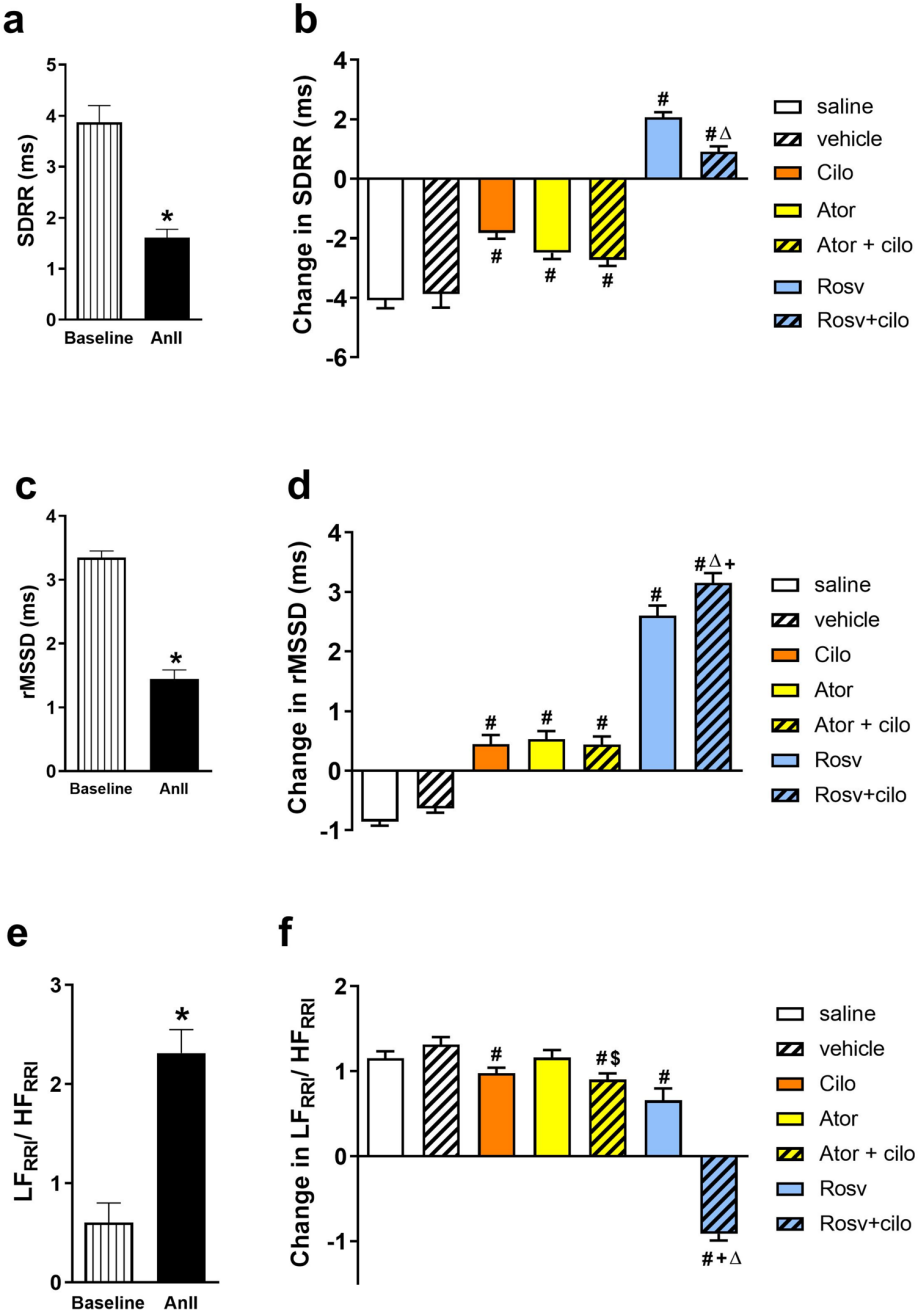
Effect of acute hypertension induced by intravenous infusion of angiotensin II (AnII) at 120 ng.min^−1^.kg^−1^ on heart rate variability demonstrated by standard deviation of beat-to-beat interval (SDRR, **a**), and root mean square of successive differences (rMSSD, **c**) and on sympathovagal balance expressed as ratio of power of beat-to-beat interval spectra in low frequency range to that in high frequency range (LF_RRI_/HF_RRI_ ratio, **e**) of adult male Wistar rats. Impact of slow intravenous injection of atorvastatin (Ator, 2 mg.kg^−1^) and rosuvastatin (Rosv, 2 mg.kg^−1^) on cilostazol (Cilo, 2 mg.kg^−1^) effect on SDRR (**b**), rMSSD (**d**), and LF_RRI_/HF_RRI_ ratio (**f**) of hypertensive rats. Values are presented as mean ± SEM (*n* = 6/group). ^*^p < 0.05 vs. baseline, ^*#*^*p* < 0.05 vs. vehicle-treated group, ^*Δ*^*p* < 0.05 vs. Cilo-treated group, ^*$*^*p* < 0.05 vs. Ator-treated group and.^+^*p* < 0.05 vs. Rosv-treated group using paired Student *t* test and one-way ANOVA followed by Bonferroni post hoc test. RRI (beat-to-beat interval)

Following intravenous infusion of AnII (120 ng.kg^−1^.min^−1^), HRV indexes (SDRR, rMSSD) were reduced as compared to baseline recordings (Fig. 5a and c). On evaluating HRV indexes following the 2 mg.kg^−1^ dose, cilostazol and its coadministration with rosuvastatin caused significant increase in SDRR and rMSSD compared to vehicle treated group (Fig. 5b and d). Interestingly, rosuvastatin, but not atorvastatin, co-injection with cilostazol exhibited greater increase in SDRR and rMSSD as in comparison with cilostazol alone (Fig. 5b and d).

### Effect on sympathovagal balance

As presented in Fig 5e, intravenous infusion of AnII (120 ng.kg^−1^.min^−1^) caused significant elevation of sympathovagal balance (LF_RRI_/HF_RRI_) ratio when compared to baseline recordings. On the other hand, the LF_RRI_/HF_RRI_ ratio was assessed following the 2 mg.kg^−1^ dose to evaluate sympathovagal balance. Cilostazol and its coadministration with rosuvastatin caused significant reduction in LF_RRI_/HF_RRI_ ratio compared to vehicle treated group (Fig. 5f). Noteworthy, rosuvastatin co-injection with cilostazol exhibited further reduction of LF_RRI_/HF_RRI_ ratio as compared to cilostazol alone indicating better vagal control of the heart, while atorvastatin co-injection caused no further reduction when co-injected with cilostazol (Fig. 5f).

### Effect on vasomodulators (serum NO and NE)

**Fig. 6:**
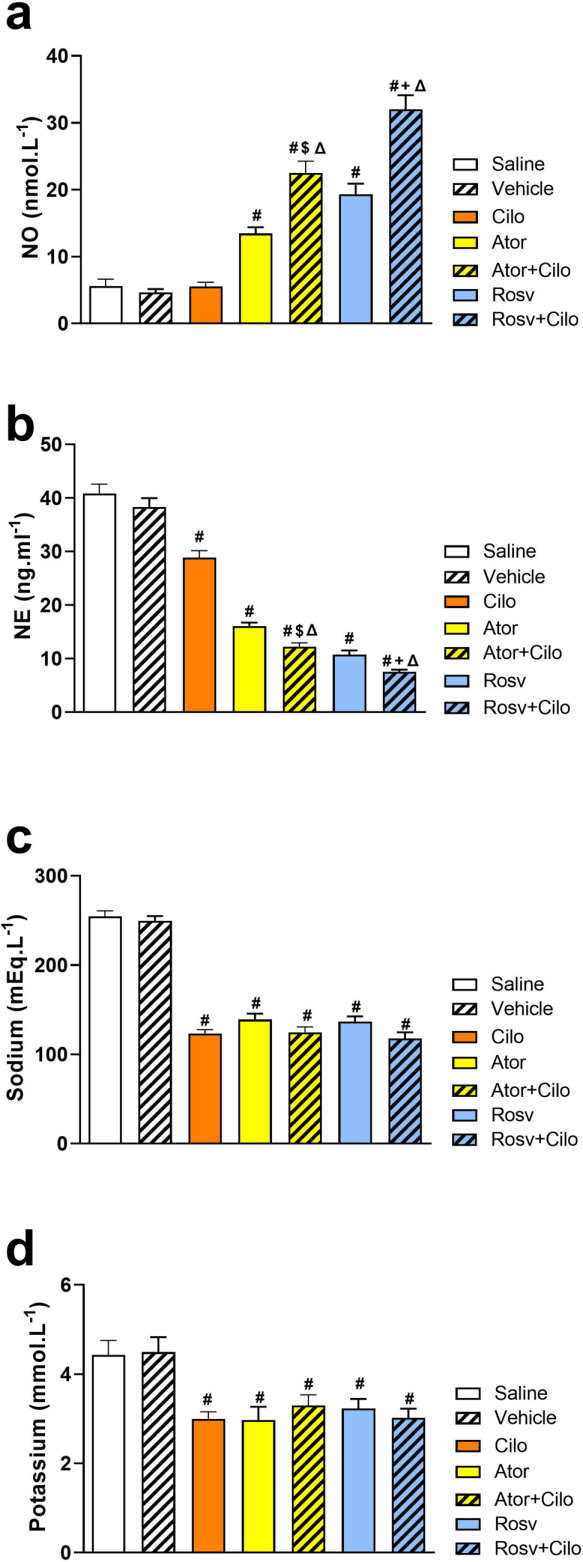
Impact of slow intravenous injection of atorvastatin (Ator, 2 mg.kg^−1^) and rosuvastatin (Rosv, 2 mg.kg^−1^) on cilostazol (Cilo, 2 mg.kg^−1^) effect on serum nitric oxide **(**NO, **a**), norepinephrine (NE,** b**) sodium (**c**), and potassium (**d**) of hypertensive adult male Wistar rats. Acute hypertension was induced by continuous infusion of angiotensin II at 120 ng.min^−1^.kg^−1^. Values are presented as mean ± SEM (*n* = 6/group). ^*#*^*p* < 0.05 vs. vehicle-treated group, ^Δ^*p* < 0.05 vs. Cilo-treated group,^*$*^*p* < 0.05 vs. Ator-treated group, and ^+^*p* < 0.05 vs. Rosv-treated group using one-way ANOVA and Bonferroni post hoc test

Cilostazol, when injected alone, failed to increase serum NO level, however, atorvastatin and rosuvastatin caused significant increase in NO level compared to vehicle group. Interestingly, coadministration of cilostazol with either statin significantly increased NO level as compared to either vehicle, cilostazol, or even statin-treated group (Fig. 6a). On the other hand, cilostazol caused a significant reduction in serum NE level compared to vehicle treated group. Noteworthy, both statins co-injection with cilostazol exhibited further reduction in NE when compared to vehicle and cilostazol alone (Fig. 6b).

### Effect on sodium and potassium

As presented in Fig. 6c and d, compared to vehicle treated group and following the intravenous injection of 2 mg.kg^−1^, cilostazol caused significant reduction of both serum sodium and potassium with greater reduction of serum sodium when co-injected with rosuvastatin.

## Discussion

Hypertensive emergency is a life threating acute elevation in BP that is typically accompanied with signs of target-organ damage (Alley and Copelin 2018; van den Born et al. 2019). To alleviate dysfunction in the affected organs, hypertensive emergency must be effectively and promptly managed through intravenous infusion of direct vasodilators; meanwhile, vasodilator-triggered reflex tachycardia may mask their antihypertensive activity and cause aortic dissection (Aggarwal and Khan 2006; Estrera et al. 2006; van den Born et al. 2019). Due to its vasodilator effects, cilostazol is frequently recommended for peripheral vascular disease. Interestingly, extensive research has highlighted cilostazol beneficial effects on the heart and cardiovascular diseases as well as its ability to reduce AnII-induced elevated SBP (Reddy et al. 2018; Sun et al. 2009; Tsuchiya et al. 2002; Z. Zhao et al. 2016). Moreover, hypertension and hyperlipidemia are major risk factors for cardiovascular diseases that often coexist (Borghi et al. 2022). Statins, such as rosuvastatin and atorvastatin, are commonly used for dyslipidemia and were reported to possess hypotensive effects beyond their hypolipidemic effect (Bautista 2009; Borghi et al. 2001; Kuklinska et al. 2010; Li et al. 2011; Susic et al. 2003).

The current study is the first to scrutinize the impact of either rosuvastatin or atorvastatin on the antihypertensive effect of cilostazol in AnII-induced hypertensive emergency in rats. This model was chosen because AnII-infusion elicits sustained hypertension, precipitates profound vasoconstriction, and engages all the cardiovascular control centers in the pathogenesis of hypertension (Badyal et al. 2003), making it a useful tool in our study. Further, RAAS is broadly activated in human hypertension, thus the chosen model resembles some forms of human hypertension can mimic hypertensive emergency in humans (Badyal et al. 2003). In attempt to understand this impact, real time invasive BP recording was performed using 3 successive doses (0.5, 1, and 2 mg.kg^−1^) for each treatment from which arterial hemodynamics and cardiac conductance were computed. Furthermore, the study investigated the effects on BRS, HRV, cardiovagal balance, vasomodulators, and electrolyte balance.

Herein, cilostazol successfully attenuated AnII-induced elevations in BP which is in accordance with previous studies that reported BP lowering effects of cilostazol (Reddy et al. 2018; Sahin et al. 2013). Similar findings were observed with both statins which are in accordance with previous studies that reported cholesterol-independent BP lowering effects of atorvastatin and rosuvastatin (Kuklinska et al. 2010; Li et al. 2011; Susic et al. 2003). The major findings herein include cilostazol’s ability to reduce elevated SBP with increased effect upon coadministration with rosuvastatin, as demonstrated by SBP reduction of 30 mmHg in just 30 min consistent with the current guidelines for hypertensive emergency management where a reduction of the average BP by 20–25% in the first hour is a requirement to alleviate end-organ dysfunction (van den Born et al. 2019). Nicardipine and nitroprusside, which are direct vasodilators commonly employed in the management of hypertensive emergencies, has been shown to reduce elevated systolic BP by 45 and 40 mmHg, respectively within the first hour of administration (Yang et al. 2004).Our findings are of a comparable reduction in raised SBP, more than 30 mmHg, within just 30 min following the co-injection of cilostazol and rosuvastatin combination accentuating the potential efficacy of this novel approach.

The antihypertensive action of cilostazol is probably achieved through a decrease in peripheral resistance, heart workload, serum sodium and potassium, reestablishment of BRS, normal HRV, sympathovagal balance, and finally the rosuvastatin-induced increase of most cilostazol effects. Peripheral resistance indexes include DBP, pulse, dicrotic-notch pressures, and SDP difference (Dart and Kingwell 2001; Giosa et al. 2021). Pulse pressure, an indicator for large arteries increased stiffness, has long been used as a strong predictor of hypertension-induced organ damage whereas DBP reflects systemic vascular resistance (Niiranen et al. 2019). Therefore, lowering pulse pressure as well as DBP and thus reducing SBP could improve arterial compliance. Dicrotic-notch pressure which reflects the pressure waveform in aorta is a common marker for ventricular ejection period end and reflects the MAP value, and therefore peripheral resistance (Balmer et al. 2021; Politi et al. 2016). On the other hand, SDP difference reflects the coupling between myocardial contractility and afterload. Collectively, antihypertensive therapies usually aim at improving arterial and ventricular stiffness, enhancing ventricular–arterial coupling, and improving left ventricle efficiency (Lam et al. 2012). In the current study, BP-lowering effects of cilostazol may be attributable to attenuation of peripheral resistance and consequently vasodilation where it successfully lowered DBP, pulse, dicrotic-notch pressures, and SDP. Interestingly, rosuvastatin added to cilostazol alleviation of AnII-induced peripheral resistance elevation which could explain the increase of cilostazol antihypertensive effect when co-administered with rosuvastatin, but not atorvastatin. The superiority of cilostazol/rosuvastatin combination over cilostazol/atorvastatin may be attributable to different effects of individual statins rather than differences in interaction; this is obvious with SBP, DBP, MAP, and dicrotic notch pressure.

Another contributor to BP is heart rate where most antihypertensive therapies reduced high BP through targeting and lowering heart rate (Reule and Drawz 2012). Herein, unlike most vasodilators, both cilostazol and rosuvastatin reduced heart rate following 2 mg.kg^−1^ dose. This, at least in part, explained its ability to reduce cardiac workload and oxygen demand (Tanna et al. 2019). Contrarily, atorvastatin exhibited reflex tachycardia and could be the reason behind atorvastatin failure to enhance cilostazol antihypertensive actions. Moreover, left ventricular ejection duration is considered a good marker for systolic function and largely affected by arterial stiffness. The duration of left ventricular ejection is decreased when arterial stiffness is markedly increased due to an increased afterload (Salvi et al. 2013). Rosuvastatin co-injection diminished ejection duration reduction induced with cilostazol demonstrating direct cardioprotective effect of such combination. To sum up, rosuvastatin added to cilostazol’s effects on cardiovascular hemodynamics exhibiting superior effectiveness compared to atorvastatin.

To better understand the effect of either statin on the antihypertensive actions of cilostazol, BRS, HRV, and sympathovagal balance which are relevant predictors for cardiovascular risk in humans were evaluated in this study. Baroreflex is a major neural regulatory mechanism of BP (La Rovere et al. 2011). Additionally, hypertensive emergency has been linked to impaired BRS and imbalance between sympathetic and parasympathetic tone (de Queiroz et al. 2013; Lagi and Cencetti 2015; Valensi 2021). Therefore, BRS-heart rate reflexes control and sympathovagal balance reestablishment is considered as antihypertensive mechanisms (de Leeuw et al. 2017; Navaneethan et al. 2009).

Baroreflex sensitivity was assessed herein utilizing parameters from sequential domain (seq BRS-SAP TOTAL) and spectral domain including LF-α and HF-α which reflect sympathetic and parasympathetic output, respectively (Shaltout and Abdel-Rahman 2005). These parameters exhibited a significant reduction following AnII infusion, indicating a notable decrease in BRS (Alipov et al. 2005; Shaltout and Abdel-Rahman 2005). In the current study, cilostazol and rosuvastatin successfully restored BRS as demonstrated by HF-α, LF-α, and seq BRS-SAP TOTAL increments. This might, in part, explain these cilostazol and rosuvastatin induced bradycardia. On the other hand, atorvastatin failed to restore BRS which explains the observed reflex tachycardia after its administration. In addition, rosuvastatin co-injection enhanced cilostazol restoration of BRS, as evidenced by further increase of HF-α and LF-α. Such improvements elicited on cilostazol co-injection with rosuvastatin, but not atorvastatin, might constitute another explanation for rosuvastatin-induced enhancement of the direct antihypertensive effect of cilostazol. However, rosuvastatin individually performed better than atorvastatin which might explain the superiority of the corresponding combination.

Further, the LF_RRI_/HF_RRI_ ratio can serve as an indicator for assessing the balance between sympathetic and parasympathetic activity, also known as sympathovagal balance. A decrease in the LF_RRI_/HF_RRI_ ratio indicates a more balanced sympathovagal state (Shaltout and Abdel-Rahman 2005). Another interesting finding in the current study is that cilostazol successfully restored sympathovagal balance depicted by LF_RRI_/HF_RRI_ ratio reduction. In addition, rosuvastatin co-injection with cilostazol exhibited greater restoration of this balance, as indicated by a further decrease in the LF_RRI_/HF_RRI_ ratio. According to this finding, rosuvastatin boosted antihypertensive activities of cilostazol, probably by contributing to the re-establishment of sympathetic/parasympathetic balance. Moreover, norepinephrine, a principal neurotransmitter of the sympathetic nervous system, is frequently assessed as an index of sympathetic activity. In conditions like heart failure, there is an observed increase in plasma NE due to increased sympathetic activity (Denfeld et al. 2018). Cilostazol demonstrated its effectiveness in mitigating increased sympathetic activity resulting from AnII infusion, as demonstrated by a notable decrease in NE levels. Cilostazol and rosuvastatin co-injection demonstrated a greater decrease in NE levels, revealing the enhancing effect of rosuvastatin on cilostazol’s effects. Moreover, sympathetic output reduction was consistent with the sympathovagal balance assessment from spectral domain analysis, indicating that the restored balance was a result of the increased parasympathetic (vagal) dominance consequent to the reduction in sympathetic output.

Furthermore, SDRR and rMSSD are two parameters used to measure HRV, and when both SDRR and rMSSD decrease, it indicates an impaired HRV (Shaltout and Abdel-Rahman 2005). Noteworthy, cilostazol has demonstrated a significant effect on the restoration of HRV presented by elevating both SDRR and rMSSD. However, an interesting finding has emerged wherein the co-administration of rosuvastatin further boosted cilostazol’s ability to restore HRV. This finding implies an additional explanation for the enhanced antihypertensive effect of cilostazol induced upon rosuvastatin co-injection.

Endothelial dysfunction, marked by reduced NO level, is a significant contributor to the risk of hypertension and cardiovascular disease. Nitric oxide plays a crucial role in controlling BP by directly influencing vascular tone. In addition, it affects vascular tone by inhibiting the sympathetic nervous system activity, both centrally and peripherally (Hermann et al. 2006). Unlike cilostazol, both statins exhibited improvements in endothelial dysfunction associated with hypertensive emergency by increasing NO level. A modulatory role of statins on eNOS and consequently NO has been reported and may have an imperative influence on ameliorating cardiovascular disorders (Balakumar et al. 2012). Interestingly, when cilostazol was administered alongside statins, it enhanced the effectiveness of statins in increasing NO level which is consistent with a previous study reported that atorvastatin and cilostazol had synergistic effect on eNOS phosphorylation and therefore NO production (Manickavasagam et al. 2007).

Furthermore, elevated serum sodium is considered as an alternative marker for hypertension-induced elevation of pulse pressure and arterial stiffness in cardiovascular events (Nowak et al. 2019; X. Zhao et al. 2018). Interestingly, treatment with either cilostazol or statins effectively reduced the elevation of serum sodium and potassium induced by AnII with greater sodium reduction when co-injected with rosuvastatin reflecting the ability to modulate extracellular volume which further adds to their antihypertensive effect.

## Conclusion

In conclusion, cilostazol alleviated high BP possibly through attenuating peripheral resistance, restored BRS, HRV, and sympathovagal balance as well as reducing heart workload, serum NE, and electrolytes together they contribute to its BP-lowering effects. Rosuvastatin, but not atorvastatin, enhanced cilostazol’s antihypertensive effects via modulating arterial hemodynamics, cardiac conductance, BRS, sympathovagal balance, and vasomodulators, which at least in part can be attributed to individual statins different effects. It is irrefutable that human hypertension pathogenesis remains obscure, and regulation of BP is multifactorial, thus generalizing study observations from a single animal model to the human circumstances is not feasible, thus it is recommended to use other animal models of hypertension to ensure the consistency of our findings before generalization. Further, investigating cilostazol, statins, and their combination for chronic hypertension is recommended.

## Data Availability

The datasets generated during and/or analyzed during the current study are available from the corresponding author upon reasonable request.

## Abbreviations

AnII: Angiotensin II
BP: Blood pressure
BRS: Baroreflex sensitivity
DBP: Diastolic blood pressure
ECG: Electrocardiogram
HF-α: Square root of the ratio of RRI and SAP in high frequency range
HRV: Heart rate variability
LF_RRI_/HF_RRI_ ratio: Ratio of power of RRI spectra in low frequency range to that in high frequency range
LF-α: Square root of the ratio of RRI and SAP in low frequency range
MAP: Mean arterial pressure
rMSSD: Root mean square of successive differences
RRI: Beat-to-beat interval
SAP: Systolic arterial pressure
SBP: Systolic blood pressure
SDP-difference: Systolic-dicrotic pressure difference
SDRR: Standard deviation of beat-to-beat interval
seq BRS-SAP TOTAL: Total sequences of at least three beats in which SAP consecutively increases or decreases accompanied by changes of the RRI of the subsequent beat in the same direction
NE: Norepinephrine
NO: Nitric oxide
